# Revealing Cell Envelope Heterogeneity in Two Stable *Escherichia coli* L-Forms

**DOI:** 10.3390/ijms27073121

**Published:** 2026-03-30

**Authors:** Boying Xu, Yueyue Zhang, Yunfei Liu, Christian Hoischen, Martin Westermann, Akiko Kashiwagi, Tatsuya Kato, Tetsuya Yomo, Jian Xu

**Affiliations:** 1 School of Ecological and Environmental Sciences, East China Normal University, Shanghai 200241, China; 2 Laboratory of Biology and Information Science, School of Life Sciences, East China Normal University, Shanghai 200062, China; 3 CF Imaging, Leibniz Institute on Aging, Fritz Lipmann Institute (FLI), Beutenbergstraße 11, 07745 Jena, Germany; 4 Center for Electron Microscopy, Medical Faculty, Friedrich Schiller University Jena, Ziegelmühlenweg 1, D-07743 Jena, Germany; 5 Faculty of Agriculture and Life Science, Hirosaki University, Hirosaki 036-8561, Japan; 6 Laboratory of Biotechnology, Green Chemistry Research Division, Research Institute of Green Science and Technology, Shizuoka University, 836 Ohya, Shizuoka 422-8529, Japan

**Keywords:** bacterial cell wall, L-form, heterogeneity, morphology, cell membrane

## Abstract

Long-term adapted cell wall-deficient (L-forms) bacteria show unique cell shapes and growth patterns compared to wild-type bacteria. However, quantitative analysis to assess the morphological heterogeneity of existing L-forms is limited. In this study, we validated that two stable L-form strains of *Escherichia coli* (NC-7 and LWF+) hold spherical or pleomorphic morphology in confocal and electron microscopy. Using imaging flow cytometry, we further reported that the variations in cell size distribution and cell viability between L-forms and walled cells are statistically significant. Moreover, freeze-fracture electron microscopy observations revealed a clear presence of an outer membrane in NC-7 but not in LWF+, suggesting that *E. coli* L-form strains could survive in both spheroplastic and protoplastic forms after adaptive evolution. Accordingly, the mutations in genes associated with cell envelope and outer membrane components are more prevalent in the LWF+ genome, potentially leading to outer membrane depletion. Notably, experimental evidence derived from *E. coli* LWF+ cells exhibiting a monoderm phenotype may support the diderm-to-monoderm transition hypothesis, implying the monoderm phenotype arose from the evolutionary loss of the outer membrane in diderm ancestors. Taken together, our findings offer insights into quantification analysis and the cell envelope status of two *E. coli* L-forms, facilitating future investigations into genotype-phenotype associations in these two L-form bacteria models.

## 1. Introduction

Bacteria can be compelled to grow and propagate in a cell wall-deficient (CWD) state, known as L-form, under stress conditions like cell wall-targeted agents, osmotic stress, nutrient starvation, cryogenic stress, and phage infection [1,2,3,4]. To date, L-form cells have been found to be prevalent in plant- and human-derived samples, encompassing various tumor microbiomes and urine samples collected from elderly individuals experiencing recurring urinary tract infections (UTI), highlighting their substantial clinical relevance [5,6,7]. The loss of the peptidoglycan layer in L-form bacteria generally leads to resistance against a broad spectrum of wall-targeting antibiotics and causes considerable pleiotropic changes in their characteristics [1,8,9]. The disrupted cell symmetry also contributes to a significant variability in both cell size and shape observed in most L-forms. In contrast to binary fission conserved in most modern bacteria, L-forms of different species (e.g., *Bacillus subtilis* and *Escherichia coli*) divide through uncovered biophysical mechanisms, similar to lipid vesicles, where changes in shape and volumes are the main triggers [10,11,12,13,14,15,16]. Although the complicated mechanism remains largely unknown, such biophysical and biological forces from both extracellular and intracellular environments of L-form cells might represent one kind of cell division fashion in early life on Earth [17]. Thus, understanding the genetic and mechanistic basis of L-forms may shed light on the minimal requirements for segregating the membrane-bounded cellular compartment essential for a living model cell or protocell in a biophysical and biological manner, providing insights into constructing a synthetic cell without a cell wall from scratch [15,17,18].

Unlike monoderm Gram-positive bacteria, the cell envelope of diderm Gram-negative bacteria comprises a protective second membrane bilayer (outer membrane, OM) that shields the peptidoglycan (PG) and effectively safeguards the cell against various environmental toxins, including antibiotics and osmotic fluctuations [19,20]. Given the diderm status of Gram-negative bacteria, it is worth arguing if there are two possible types of stable L-forms, i.e., spheroplast and protoplast, in Gram-negative bacterial species like *E. coli* (Figure S1A). In *Proteus mirabilis* (Gram-negative), the considerable differences in the protein pattern of isolated membrane proteins and their L-forms, both the unstable spheroplast L-form (UL19) and the protoplast L-form (LD 52), have been recorded previously [21]. There are two stable L-forms available from *E. coli*, named LWF+ and NC-7, which were generated via long-term adaptation in sole Penicillin G (PenG) and the combination of mutagen *N*-Methyl-*N*′-nitro-*N*-nitrosoguanidine (MNNG), lysozyme (peptidoglycan *N*-acetylmuramoylhydrolase), and PenG, respectively ([22,23], Figure S1B). Regarding the cell envelope components, it has been reported that the LWF+ is a protoplast-type L-form [24], lacking the typical components of the Gram-negative envelope, especially the OM, as shown in transmission electron microscopy (TEM) [22,25]. Osawa & Erickson (2019) [26] identified the *E. coli* NC-7 as a spheroplast-type of L-form through observations of its cell tubulation property and membrane staining techniques. The outer membrane in NC-7 cells was crucial in maintaining angular shapes and resisting turgor in low-osmolality media [26]. Previously, the comparative genomics of these two L-forms revealed both unique and common mutated genes, many of which belong to essential gene categories not involved in cell wall biosynthesis [27]. Therefore, one might inquire as to the existence of particular disrupted genetic networks associated with the differing cell envelope phenotypes observed between the two L-forms.

In the current study, the quantitative analysis of NC-7 and LWF+ strains using imaging flow cytometry (IFC) was performed to evaluate the morphological heterogeneity of the two L-forms cultured in the liquid medium. We showed that the variations in cell size distribution and cell viability between two L-forms and walled cells are statistically significant. The cell shape and envelope status were then confirmed by various light and electron microscopy techniques, from which a clear presence of an outer membrane in NC-7 but not in LWF+ was observed, suggesting that *E. coli* L-form strains could survive in both spheroplastic and protoplastic forms after adaptive evolution. We also found a clear association between the mutated genes and cell envelope and outer membrane components in both L-forms. Taken together, our findings offer new quantitative results of two *E. coli* L-forms, facilitating future investigations into genotype-phenotype associations in these two L-form bacteria models.

## 2. Results

### 2.1. E. coli L-Form Strains Show a Noticeable Heterogeneity in Morphology

**Figure 1:**
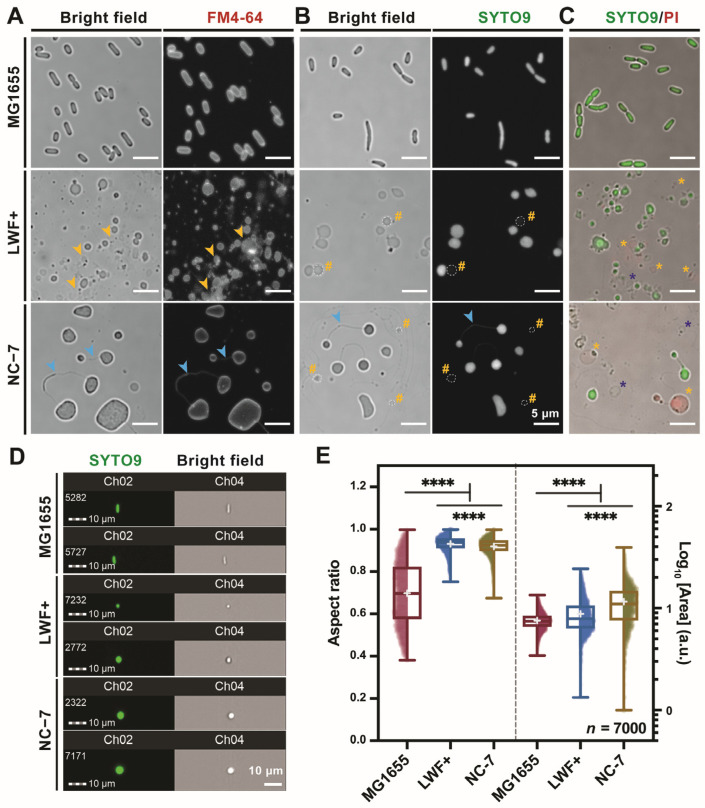
**Heterogeneity in morphology and viability in two *E. coli* L-forms, NC-7 and LWF+.** The morphology of the cells, including the wild-type *E. coli* MG1655 strain (walled cells), was confirmed by confocal microscopy stained either by membrane dye FM4-64 (**A**), nucleic acid SYTO9 (green fluorescence, viable cells, (**B**), or SYTO9 and PI (red fluorescence, dead cells) double staining (merged in (**C**)). Yellow arrowhead indicates the membrane segments generated during the cell culture of LWF+. Blue arrowhead marks the stalk structure connecting the daughter cells in NC-7 during cell division. The SYTO9-insensitive cells and PI-stained cells in LWF+ and NC-7 cells are labeled with yellow # and *, respectively. Additionally, blue asterisks (*) denote “Ghost” cells that lack DNA staining signals. Scale bar = 5 μm. The walled wide-type *E. coli* MG1655 strain and two L-form strains were stained with SYTO9 (viable cells) and subjected to imaging flow cytometry (IFC). Representative cells with a typical aspect ratio from IFC were selected and plotted in (**D**). Scale bar = 10 μm. The boxplots with all individual data points (scatter plot showing data distribution) for the aspect ratio and the mean logarithmic area are shown for the cell populations (*n* = 7000) of MG1655, LWF+, and NC-7. Mean values from the gated population were statistically compared between each strain and the wild-type MG1655 using Welch’s *t*-test (two-tailed) (**E**). **** indicates a significance with *p* value of <0.0001.

Based on confocal microscopy images, it was observed that LWF+ and NC-7 strains exhibited a complete loss of rod-shaped morphology, displaying distinct variations in both size and morphology when compared to the wild-type *E. coli* MG1655 cells (WT, Figure 1A, upper panel), as previously documented in earlier studies [23,25]. Specifically, LWF+ cells exhibited a spherical shape with a relatively small diameter (~1 μm), whereas NC-7 cells displayed pleomorphic and asymmetrical forms, with some cells being significantly larger than both LWF+ and MG1655 cells. When subjected to cell membrane staining with the membrane dye FM4-64, both L-forms exhibited a substantial presence of membrane segments, possibly from the unsuccessfully divided dead cells or outer membrane vesicles (OMVs), since Gram-negative bacteria produce OMVs that are shed from the outer membrane during cell growth [28]. There are many visible granules, vacuoles, and other vesicle-like structures similar to ghost membrane death cells in LWF+ cells (Figure 1B, middle panel, yellow hash #), as well as cell stalk-like membrane tubes connecting multiple sister cells found in NC-7 cells (Figure 1B, lower panel, blue arrowheads), both of which are absent in WT cells. To further evaluate the viability of these L-form cells, we then utilized the Live/Dead (SYTO9/PI) bacterial cell staining method. The results depicted in Figure 1C demonstrate a proportion of L-form cells exhibiting sensitivity to PI (Figure 1C, yellow asterisk *), confirming reduced cell viability in L-form cells following prolonged adaptation. Additionally, we also observed that some cell-like structures are not sensitive to either of the nucleic acid dyes (Figure 1C, blue asterisk *), indicating that L-forms could produce nucleic acid-less membrane vesicles during cell growth.

### 2.2. Statistical Analysis of Cell Morphology of L-Forms by Imaging Flow Cytometry

**Figure 2:**
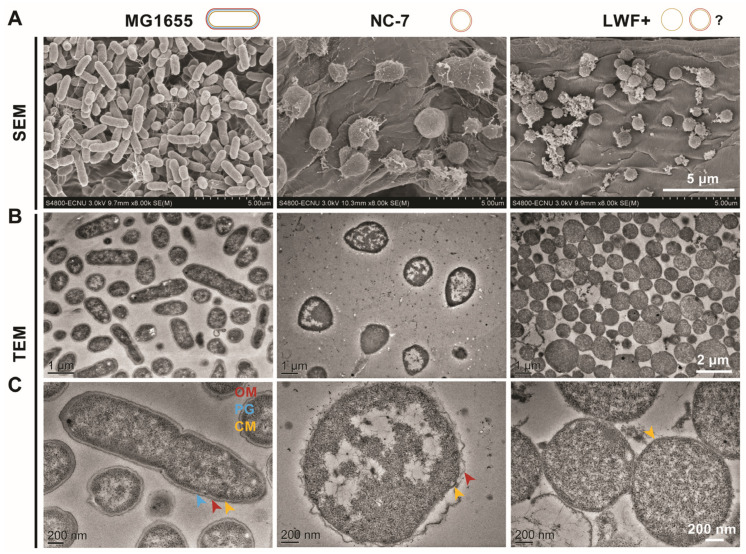
**Electron microscopy of *E. coli* L-forms.** Scanning electron microscopy (SEM, (**A**)) and transmission electron microscopy (TEM, (**B**)) of the walled strain MG1655 (**left panel**), *E. coli* L-form strain NC-7 (**middle panel**), and LWF+ (**right panel**) were shown. Cell envelope structures were further examined in the magnified TEM images (**C**). Unlike MG1655, both L-forms lack a peptidoglycan layer. NC-7 retains both inner and outer membranes, as protrusions are often visible from the outer membrane. In contrast, for LWF+, it remains unclear whether an outer membrane is present, since only one membrane was detected. OM: outer membrane, red arrowhead; PG: peptidoglycan, blue arrowhead; CM: cytoplasmic membrane, yellow arrowhead. The scale bar for each image is indicated.

IFC-based analysis enables simultaneous visualization of individual cell images and quantitative parameters, facilitating image-guided gating for precise and high-quality data selection [29]. We then aim to perform a quantitative analysis of several key cell characteristics (e.g., aspect ratio and area) of the L-forms, given their significant morphological fluctuations. As validated by the above Live/Dead cell staining approaches, SYTO9 could distinguish and stain live cells out of all L-forms when both SYTO9/PI dyes are present. Both SYTO9-stained L-form cells were then subjected to IFC analysis, and two representative single-cell images for each cell type are shown in Figure 1D. The quantitative IFC results are from ~7000 single cells as plotted in Figure 1E, including the mean value and distribution of the aspect ratio and area. It reveals that the aspect ratio (mean ± standard deviation (SD)) of LWF+ (0.93 ± 0.04) and NC-7 (0.92 ± 0.04) live cells is significantly higher than that of MG1655 (0.70 ± 0.15), indicating that the two L-forms are more spherical. As expected, the area (mean ± SD) among the three strains is also remarkably different (MG1655: 7.32 ± 1.41, LWF+: 7.45 ± 2.38, and NC-7: 10.60 ± 5.23). It is worth noting that the apparent discrepancy between IFC and confocal microscopy may stem from differences in cell-size capture. In IFC measurements, both large and small cells are included, whereas confocal microscopy may preferentially visualize larger cells owing to technical limitations. Consequently, L-form cells may not appear significantly larger than wild-type cells based on the mean values in the IFC dataset. These results showed that both LWF+ and NC-7 are in a bigger size and distributed in a broader range (reflected by the scatter plot) than WT cells, which are consistent with the results of confocal microscopy, as shown in Figure 1A–C, and SEM images (Figure 2A).

### 2.3. LWF+ Shows Monoderm Phenotype Confirmed by Electron Microscopy

The cell shape was also validated in either SEM (Figure 2A) or TEM (Figure 2B,C) with improved details. It is apparent from SEM images that both strains are in non-rod shapes, and a higher level of deformation was observed in NC-7 cells compared with LWF+. The cell wall deficiency can be recognized in TEM images where both strains have no peptidoglycan layer (blue arrowhead in MG1655 cells, Figure 2C, left panel) present on the cells. As shown in Figure 2B (middle panel), NC-7 L-forms retain both inner (CM, yellow arrow) and outer membranes (OM, red arrow), as some protruding structures can frequently be visible from the outer cell membrane, which has also been confirmed in a recent report [26]. In contrast, unlike the typical double-membrane structure of NC-7, it is still elusive to conclude if LWF+ holds an outer membrane since only one membrane could be identified (Figure 2B, right panel), which is consistent with the statement made on the LWF+ strain in the earlier literature [24,25]. However, it does not rule out the possibility that the distance between the two membranes is too close to be distinguished in LWF+.

**Figure 3:**
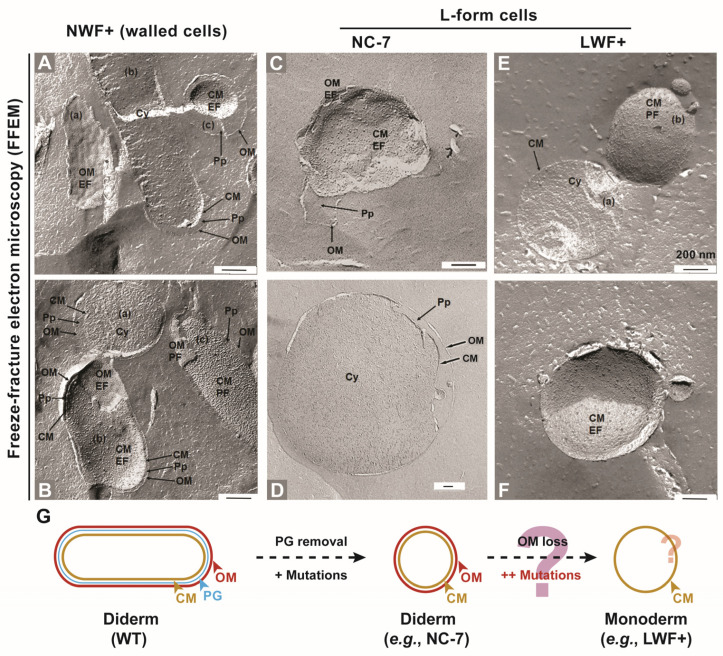
**Freeze-fracture electron microscopy of the cell-walled *E. coli* NWF+ and the L-form NC-7 and LWF+.** Cytoplasmic membrane CM, outer membrane OM, and periplasm Pp are labeled by black arrows. Black scale bars indicate 200 nm. (**A**) Three cells of *E. coli* NWF+ (wall cells); the cell on the left (**a**) shows the exoplasmic fracture face EF of the OM, the cell in the middle (**b**) is cross-fractured, and small areas of CM, Pp, and OM are visible at one cell pole. The cell on the right (**c**) is fractured at the cell pole in the longitudinal axis and offers a view of the EF face of the CM, Pp, and cross-fractured OM. (**B**) Three cells of *E. coli* NWF+; the cell on the top (**a**) is cross-fractured. The cell on the left (**b**) shows the EF face of the OM and of the CM. The cell on the right (**c**) shows the protoplasmic fracture face (PF) of the OM and of the CM. (**C**) Cell of the *E. coli* NC-7 L-form with the EF face of OM (small area) and CM, the Pp, and the OM. (**D**) Cross-fracture of a large *E. coli* NC-7 L-form cell with a view of the cytoplasm, CM, Pp, and OM. (**E**) Two cells of *E. coli* LWF+; the cell on the left (**a**) is cross-fractured through the cytoplasm Cy, and the cell on the right (**b**) is membrane fractured, showing the PF face of the CM. Structures of the OM are not detectable. (**F**) Cells of *E. coli* LWF+; shown is the EF face of the CM. The structures of the OM are not detectable. The possible transition processes in two L-form strains, either spheroplasts (diderm) or protoplasts (monoderm), are indicated in (**G**). The unknown or uncertain aspects are marked with a question mark (?).

According to Allen et al. [1], one can distinguish between spheroplast-type L-forms containing the cytoplasmic membrane (CM) and some structures of the cell wall (in most cases, the OM) and protoplast-type L-forms, which are surrounded only by the CM and contain no visible structures of the cell wall. Both types can again be categorized into stable L-forms, which cannot revert anymore into the normal walled parental bacteria, and into the unstable L-forms, which can revert. We employed freeze-fracture electron microscopy (FFEM) in order to show large and detailed ultrastructural views of cellular membranes. We therefore compared the cell-walled parental strain NWF+, which is derived from *E. coli* MG1655, with its stable *E. coli* L-form LWF+ as well as with the stable *E. coli* NC-7 L-form. The analysis of the rod-shaped NWF+ offers many views of the cell wall structures, i.e., outer membrane OM and periplasm Pp, which are easily detectable. In Figure 3A,B are some examples of cross fractures with clearly distinguishable CM, Pp, and OM. Fractures of the CM with exoplasmic fracture face (EF) and protoplasmic fracture face (PF), and fractures of the OM with EF and PF can also be observed. The inner membrane CM and the outer membrane OM can be distinguished by a step in the freeze-fracture plane, and EF and PF of the OM can be identified by the concave shape EF and the intramembrane particles pattern caused by protein-lipopolysaccharide complexes, or the convex shape PF of the fracture faces [30].

We then carefully examined the electron micrographs of the spherical *E. coli* LWF+, and, besides the CM, we never observed any structures or traces of the OM. One typical cell (a) from Figure 3E shows a cross-fracture of LWF+ through the cytoplasm with a clearly visible CM and no detectable OM or periplasm. Other typical cells in Figure 3E cell (b) and Figure 3F are seen with fractures of the cytoplasmic membrane with a protoplasmic fracture PF face as well as an exoplasmic fracture EF face. EF and PF of the CM can be identified by the shape of the fracture face. The exoplasmic fracture face of the cytoplasmic membrane is characterized by a concave shape and a low density of intramembrane protein particles, whereas the protoplasmic fracture face shows a convex shape and a high density of intramembrane protein particles [31]. However, an outer membrane OM and a periplasm Pp are clearly visible in the NC-7 L-form as shown in Figure 3C, showing fractures of the CM and of the OM, whereas cross fractures can be seen in Figure 3D with clearly visible OM and Pp. One has to take into consideration that residual small amounts of peptidoglycan would not be visible with freeze-fracture electron microscopy of L-form cells.

As illustrated in Figure 3G, these results imply that L-form may exist in multiple forms, either in spheroplast (diderm type, NC-7) or protoplast (monoderm type, LWF+), for Gram-negative bacteria, such as *E. coli*. Taken together, those significant differences between these two L-form strains indicate that there might be considerable genomic DNA variants and some distinct mutations in genes involved in bacterial size determination, membrane synthesis, and/or cell division during induction and adaptive culture in LWF+ [24] and NC-7 [26].

### 2.4. Gene Mutations Related to Outer Membrane Biosynthesis in L-Form Strains

To determine the possible association between these mutations and cell membrane biosynthesis, especially focusing on the outer membrane, we then investigated mutated genes (focusing on coding regions only) in these two L-forms reported in our previous study [27]. The results from GO analysis in Table S1 (NC7) and S2 (LWF+) reveal that genes associated with diverse cellular pathways, including protein secretion, membrane, membrane protein, cell wall, peptidoglycan biosynthesis, lipopolysaccharide, and cell envelope, underwent considerable mutations and significant enrichment, suggesting that the components of the cell envelope were extensively altered in both L-forms during adaptations.

**Figure 4:**
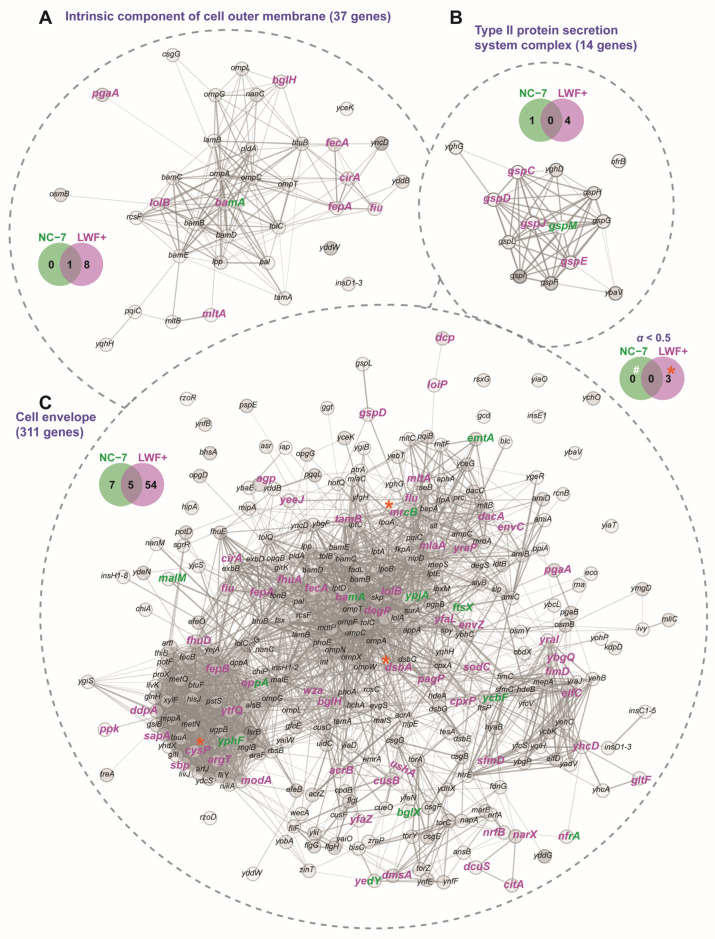
**Accumulated mutations in genes related to the cell envelope and outer membrane components.** Mutated genes detected in two *E. coli* L-forms were mapped to the function categories from the intrinsic component of the outer cell membrane ((**A**), 37 genes in total), type II protein secretion system complex ((**B**), 14 genes in total), and cell envelope ((**C**), 311 genes in total). The green and red colors indicate mutated genes found in NC-7 and LWF+, respectively. Same genes, such as *bamA*, *mrcB*, *cysP*, *oppA*, *nfrA*, and *yedY,* were shown in green-red mixed colors. The missense variants of *dsbA*, *mrcB*, and *cysP* in LWF+ were predicted to be deleterious based on the calculated mutation effects (*α* < 0.5). “#/*” indicates the number of genes found mutated in NC-7/LWF+, respectively.

The comparison between the two L-forms, as depicted in Figure 4, demonstrated that more genes associated with the intrinsic element of the outer cell membrane (Figure 4A, LWF+:NC7 (1:9), one overlapped: *bamA*), type II protein secretion system complex (Figure 4B, LWF+:NC7 (1:4), no overlapped), and cell envelope (Figure 4C, LWF+:NC7 (12:59), five overlapped: *bamA*, *mrcB*, *yedY*, *nfrA*, *oppA*) in LWF+ are found to have mutations. Notably, the missense variant effect predictions suggest that three SNPs in DsbA^Ser152Phe^ (Thiol:disulfide oxidoreductase), CysP^His97Tyr^ (Thiosulfate/sulfate ABC transporter periplasmic binding protein), and MrcB^Pro656Leu^ (Peptidoglycan glycosyltransferase/peptidoglycan DD-transpeptidase, also known as PBP1b) are highly deleterious (*α* < 0.5, Table S3) [27,32]. As a key component of the disulphide bond machinery in *E. coli*, DsbA functions as a thiol disulfide oxidoreductase within the periplasmic space, which is crucial for correct protein folding, bacterial virulence, and adaptation to oxidative stress [33,34]. The *cysP* gene in bacteria encodes a protein that is typically involved in the transport of sulfate or thiosulfate across the cell membrane, and plays a key role in the sulfate assimilation pathway, which is critical for cysteine biosynthesis and sulfur metabolism in bacteria [35,36]. The PBP1b protein exhibits both glycosyltransferase (for linking sugar chains) and transpeptidase (for cross-linking peptide chains) activities for synthesizing and maintaining the peptidoglycan layer of the cell wall, making it essential for proper bacterial morphology and survival [37,38]. The disruption or dysfunction of these genes may collectively increase the possibility that the LWF+ strain sustains more severe cell membrane disturbances, particularly of the outer membrane, as compared to the wild-type and NC-7 strains. These findings further align with the morphological analysis obtained via microscopy (Figure 1A) and TEM images (Figure 2 and Figure 3) that LWF+ may completely lose its outer membrane and exhibit a monoderm phenotype.

## 3. Discussion

In this study, significant cell size and morphology differences were observed in *E. coli* NC-7 and LWF+ L-forms, which were confirmed by microscopy as shown in Figure 2. The spherical shape of LWF+ is consistent with several early observations [22,25]. In contrast to the spherical shape of NC-7 at the time of its generation, our observation results and those of Osawa et al. showed that the NC-7 cells were pleomorphic and angular, with only a minority of spherical shapes [23,26]. We speculate that new mutations related to cell shape arose and accumulated during long-term culturing, which correlated with its morphological changes. As shown in Figure 2C and Figure 3, the images from TEM and freeze-fracture electron microscopy validated that the PG layer is not present in both L-forms, although the confirmation for the outer membrane shows an inconsistency that NC-7 has both inner and outer membranes, while LWF+ seems to only have one membrane layer. Since it is challenging to confirm the presence of an outer membrane of LWF+ from direct observation, some other direct or indirect approaches may be required to further confirm the membrane status in LWF+. Osawa et al. claimed that the outer membrane of NC-7 plays a role in maintaining its pleomorphic morphology through experimental results by adding LPS inhibitor CHIR-090 to block the synthesis of the outer membrane or adding EDTA to chelate divalent cations on the outer membrane, which changed the morphology of NC-7 from pleomorphic to spherical [26], indicating that a double membrane surrounds NC-7. Although LWF+ shows a monoderm phenotype confirmed by FFEM (Figure 3E,F), it is still too early to draw any conclusions to exclude the essentiality of an outer membrane for the formation and viability of *E. coli* L-forms [39].

Mutations in genes associated with the cell envelope and outer membrane can disrupt key pathways required for outer-membrane biogenesis and stability, ultimately leading to membrane loss under stress or during L-form transition. BamA, the principal component of the β-barrel assembly machinery (BAM complex), mediates the insertion and folding of outer-membrane proteins; defects in *bamA* weaken the protein network that underpins membrane integrity and permeability control [40,41]. LolB acts as a lipoprotein receptor and anchoring factor that ensures proper lipoprotein incorporation into the outer membrane. Impaired *lolB* function disturbs lipoprotein localization and lipid asymmetry, resulting in the destabilization of membrane structure [42,43]. MltA, a membrane-bound lytic transglycosylase that facilitates peptidoglycan remodeling and mediates coupling between the peptidoglycan layer and the outer membrane, when disrupted, causes envelope weakening and subsequent membrane detachment [44,45]. Collectively, mutations in these envelope-related genes may act synergistically to compromise peptidoglycan–outer-membrane linkage, providing a mechanistic basis for outer-membrane loss and the emergence of L-form phenotypes. Besides these examples, numerous other mutations have been detected in both L-forms [17]. However, the key genetic determinants responsible for initiating or driving outer-membrane loss remain unclear. Further investigations are required to identify which specific gene mutations are correlated with the loss of the outer membrane in LWF+ cells, particularly during or after cell division [46,47]. This uncertainty represents one of the main limitations of the present study and highlights the need for comprehensive genomic and functional analyses to elucidate the molecular mechanisms underlying outer-membrane disassembly.

Gram-positive bacteria are usually defined not only by the absence of an outer membrane but also by the presence of a thick peptidoglycan layer, whereas in the present L-form strains, the peptidoglycan layer is also missing (Figure 2). The hypothesis of the diderm-to-monoderm transition suggests that certain Gram-positive (monoderm) bacteria originated from Gram-negative (diderm) ancestors by losing the outer membrane [48]. The evolutionary scenario is reinforced by a bimodal distribution of OM-tethering systems (such as OmpM, Pal, and OmpA) across the deepest bacterial clades (Terrabacteria, Gracilicutes) [49,50]. To date, this evolutionary scenario has only been experimentally tested in the diderm Firmicutes *Veillonella parvula*, showing that disruption of OM tethering (e.g., OmpM) can significantly lead to membrane detachment reminiscent of monoderm cell architecture [51]. We also found a series of genes involved in the envelope and outer membrane biogenesis (e.g., BamA [52]) in both L-forms (Tables S1 and S2, and Figure 4). As discussed above, it is still unclear which mutated gene(s) contribute to the OM depletion observed in LWF+ cells. Nevertheless, the observed monodermic state of viable LWF+ cells should be considered experimental evidence in *E. coli*, thereby potentially supporting the transition from a diderm to a monoderm envelope structure in bacteria. We should emphasize that our observations represent a functional analogy to monoderm organization rather than direct evidence of evolutionary transformation.

The two *E. coli* K-12 strain-derived stable L-form strains, LWF+ and NC-7, were induced under different conditions (Figure S1A,B) described previously [22,23]. Besides the common β-lactam inducer PenG, lysozyme, and mutagen MNNG have been used in adapting the NC-7 strain [23]. Following the ingredients from the original literature, we successfully cultured both two L-form strains in either solid or liquid medium ([22,23]; see also materials and methods). In this study, LWF+ was cultured with shaking in BHI medium without supplements of horse serum, yeast extract, and osmotic stabilizer [24], while with NC-7, the osmoprotective MLB medium containing 340 mM NaCl was used [26] for static culture without shaking. All cultures in solid and liquid culture were shown in Supplemental Figure S1C. The growth curve showed that both L-form strains can proliferate in their pre-designed culture liquid medium, where the maximum concentration (as indicated by OD_600_) of LWF+ is higher than that of NC-7 (Figure S1D). Together with previously published literature on the culture of L-form bacteria, the restricted culture conditions in complex media are one obstacle to studying available L-form strains at both fundamental and applied levels [18,23,26,53,54,55]. Moreover, it is noteworthy to share our experience that we failed to culture and adapt both L-forms in various component-defined liquid media, such as the M63 medium supplemented with amino acids and 340 mM NaCl as an osmotic stabilizer, suggesting that some unknown substances are additionally required for stimulating the growth and/or viability of LWF+ and NC-7 L-forms by decreasing the cellular levels of reactive oxygen species (ROS) [56,57]. It is then also essential to acknowledge that the diverse culture conditions employed for all the tested *E. coli* strains, both wild-type and L-forms, render these results potentially incomparable, a limitation that must be considered within the current investigation. To advance the use of *E. coli* L-form bacteria in synthetic biology, it is crucial to establish strains that can be readily propagated in a simplified or defined synthetic medium [58].

In contrast with L-forms generated through genetic manipulations of a few key players, both L-form strains studied show significant genomic mutations after prolonged exposure to bactericidal antibiotics [59]. Although the experimental verification of the effect of each SNP is impossible, these L-forms can be employed as valuable genetic network models that are largely impaired for identifying and exploring essential components for synthetic genomes and artificial cells in a top-down manner [27,60,61,62]. It has also been observed that both L-forms produce a significant amount of cell-derived vesicles, perhaps generated during or after cell divisions, which renders them a suitable application platform for cell fusion-division analysis and protein display via further bioengineering [28,63,64,65]. Currently, we cannot ignore the limitations that additional L-forms should be established through similar approaches to further validate the reproducibility regarding the genotypes and phenotype from these two *E. coli* L-forms.

## 4. Materials and Methods

### 4.1. Bacterial Strains and Culture Conditions

The cell-walled parental strain *E. coli* NWF+ (N-form) and stable L-form *E. coli* LWF+ derived from *E. coli* K-12 W1655 F was gifted by Dr. Christian Hoischen (Fritz–Lipmann–Institute, Jena, Germany) [24,25]. Another stable *E. coli* L-form, NC-7, derived from *E. coli* K-12 3301, was originally established by Onoda et al. [23] and was gifted by Dr. Akinobu Oshima (Shimane University, Matsue, Japan). *E. coli* K-12 strain MG1655 was maintained by our laboratory (ATCC #700926) [66]. The culture conditions for all *E. coli* strains were used as reported in our previous study [27]. Briefly, *E. coli* wild type (WT) MG1655 was grown in Luria–Bertani (LB) broth (1% tryptone, 0.5% yeast extract, 170 mM (1%) NaCl). L-form LWF+ was grown in brain heart infusion (BHI) broth containing 100 U/mL Penicillin G (PenG), and NC-7 was grown in MLB medium (1% peptone, 0.5% yeast extract, 30 mM glucose, 340 mM NaCl, 1 mM CaCl_2_, 25 mM MOPS, pH 7.0) containing 100 U/mL PenG. All bacteria except NC-7 were incubated at 37 °C with shaking (200 rpm) while NC-7 cells were incubated statically without shaking at 30 °C. Growth from liquid culture (each 3 mL) under the above optimal conditions was monitored by measuring the absorbance at 600 nm, and readings with a UV–Vis spectrophotometer (T6, Persee Co., Beijing, China) were obtained at 1 h intervals for 30 h.

### 4.2. Confocal Microscopy

Live-cell imaging of cultured bacterial cells (L-forms and MG1655) was performed using an imaging spacer (Sigma, St. Louis, MO, USA) on a confocal microscope (Nikon C2plus, Yokohama, Japan). The cell membrane and nucleic acid were labeled with FM4-64 (20 μg/mL, D1306, ThermoFisher, Waltham, MA, USA) or DAPI (10 μg/mL, D1306, ThermoFisher), respectively. Live and dead cells were confirmed by LIVE/DEAD^TM^ BacLight^TM^ (SYTO^TM^9/PI, 5 μg/mL/40 μg/mL, L34856, ThermoFisher). The images from bright, blue (461 nm), green (488 nm), and red (560 nm) fluorescent channels were captured for subsequent analysis.

### 4.3. Imaging Flow Cytometry

The *E. coli* cells were analyzed using an Amnis^TM^ ImageStream^TM^X imaging flow cytometer equipped with INSPIRE acquisition software (Luminex, Austin, TX, USA). To detect green fluorescent signals from SYTO9-stained live cells, a 20 mW 488 nm laser was utilized, and the emitted light was detected using a 505–560 nm filter (Channel 2). Channel 4 was used to collect images from bright field, while side scatter (SSC) was generated using a 2 mW 785 nm laser and collected with a 745–800 nm filter (Channel 6). All images were acquired with a magnification of 40-fold (40× objective), a pixel size of 0.25 μm^2^.

Approximately 10,000 cells (data points) were obtained and subjected to gating based on fluorescence intensity and fluorescence aspect ratio intensity to exclude the internal beads and cell culture debris. Subsequently, the gating process and detailed analysis are performed using IDEAS software (v.6.2.183.0, Luminex, USA), which was detailed in Supplemental Figure S2. The cells (*n* = 7000) employed for the analysis of morphology were subsequently sorted based on the quality of sharpness in their respective images, specifically the fluorescence gradient RMS (root mean square for image sharpness), as previously outlined [29,67]. Only cell images that were in focus were included in the analysis. The major and minor axis lengths, which corresponded to the longest and narrowest dimensions of the cell image, respectively, were employed to represent the relative lengths and widths of the cells. The aspect ratio, defined as the ratio of the minor axis length to the major axis length, served as a representation of the cell shape and provided insight into the sphericity of the cell in the image. The relative cell size was assessed through simulated area (*A*), which was determined by quantifying the total number of pixels within the cell image.

### 4.4. Scanning Electron Microscopy (SEM) and Transmission Electron Microscopy (TEM)

Bacteria at the late exponential phase were centrifuged for 10 min at 5000× *g* at 4 °C, followed by washing with osmoprotective M63 medium (22 mM glucose, 1 mM MgSO_4_, 0.015 mM vitamin B1, 2 g/L (NH_4_)_2_SO_4_, 13.6 g/L KH_2_PO_4_, 0.5 mg/L FeSO_4_·7H_2_O, 340 mM NaCl) for 2 times, and fixed in 2.5% (*v*/*v*) glutaraldehyde at 4 °C. The dehydration, embedding, sectioning, and staining were performed according to our previous protocols [29]. The prepared samples were visualized using a scanning electron microscope (Hitachi S-4800, Tokyo, Japan) at an accelerating voltage of 3 kV or transmission electron microscopy (Hitachi HT-7700, Tokyo, Japan) at an accelerating voltage of 100 kV.

### 4.5. Freeze-Fracture Electron Microscopy (FFEM)

Freeze-fracturing and electron microscopy were performed following our previous protocols [68]. Briefly, at a temperature of 30 °C, walled (NWF+) and L-form (NC-7 and LWF+) cells were separated from the growth medium, washed, and resuspended in 0.4 M sucrose (10% of the initial volume). Aliquots were enclosed between two 0.1 mm copper profiles as used for the sandwich double-replica technique. The sandwiches were rapidly frozen in liquid propane and cooled using liquid nitrogen. Freeze-fracturing was performed in a BAF400T freeze-fracture unit (BAL-TEC, Balzers, Liechtenstein) at −150 °C using a double-replica stage. The samples were shadowed without etching with 2–2.5 nm Pt/C at an angle of 35°. In a second evaporation step, a carbon layer of about 20 nm thickness was deposited for mechanical stabilization from a perpendicular direction on the Pt/C replicas. The obtained replicas were transferred into a “cleaning” solution (commercial sodium hypochlorite containing 12% active chlorine (Cl_2_)) for 15 min at 45 °C. Subsequently, the replicas were washed four times in distilled water and transferred onto Formvar-coated grids for examination in a Zeiss EM 902A electron microscope (Zeiss, Oberkochen, Germany) using a 1 kV FastScan-CCD-camera (TVIPS camera and software, Munich, Germany). The electron microscopic images of freeze-fracture replicas were rotated for presentation in a way that the direction of the dark appearing Pt shadowing (35°) is approximately in the direction from bottom to top.

### 4.6. Bioinformatic Analysis of Gene Mutations

The full genome re-sequencing data for LWF+ and NC-7 L-forms were used according to our previous report under BioProject with accession number PRJNA905352 [27]. The gene ontology (GO) enrichment analysis was performed by clusterProfiler v.4.2.2 [69]. The association of genes was analyzed and visualized in STRING (version: 12.0, https://string-db.org (accessed on 25 March 2025)) [70]. The variant effect scores (*α*), predicted by the DeepSequence pipeline [32], for all missense variants were included in our previous report [27]. A high probability of loss-function mutations was considered when the *α* value is lower than 0.5 (*α* < 0.5: high; 0.5 < *α* < 0.8: medium; 1.0 < *α* > 0.8: low). The investigation in this study focused only on gene mutations related to cell envelope and outer membrane biosynthesis.

## Data Availability

The original contributions presented in this study are included in the article/Supplementary Materials. Further inquiries can be directed to the corresponding authors.

## Supplementary Materials

The following supporting information can be downloaded at: https://www.mdpi.com/article/10.3390/ijms27073121/s1.

## Abbreviations

The following abbreviations are used in this manuscript:

L-form	Cell wall-less (-deficient) bacteria
CWD	Cell wall-deficient
UTI	Urinary tract infections
PenG	Penicillin G
MNNG	*N*-Methyl-*N*′-nitro-*N*-nitrosoguanidine
TEM	Transmission electron microscopy
SEM	Scanning electron microscopy
FFEM	Freeze-fracture electron microscopy
IFC	Imaging flow cytometry
OM	Outer membrane
CM	Cytoplasmic/Inner membrane
PG	Peptidoglycan
OMVs	Outer membrane vesicles
EF	Exoplasmic fracture face
PF	protoplasmic fracture face
Pp	Periplasm
GO	Gene ontology
