# Supplementary material for: Revealing Cell Envelope Heterogeneity in Two Stable Escherichia coli L-Forms
Source: Int J Mol Sci. 2026 Mar 30;27(7):3121. doi: 10.3390/ijms27073121 (PMC13074047; doi:10.3390/ijms27073121)
Supplement: Supplementary file 1 [file ijms-27-03121-s001.zip › Fig. S1.pdf]

Fig. S1

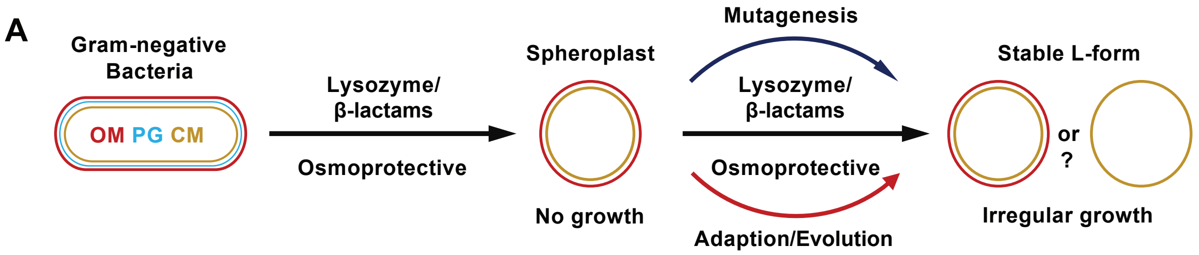

B

Strain	Origin	Transform method	Genome sequence
E. coli LWF+	*E. coli* K12 W1655 F+	Penicillin G	Full
E. coli NC-7	*E. coli* K12 3301	N-Methyl-N'-nitro-N-nitrosoguanidine (MNNG); Lysozyme; Penicillin G	Full

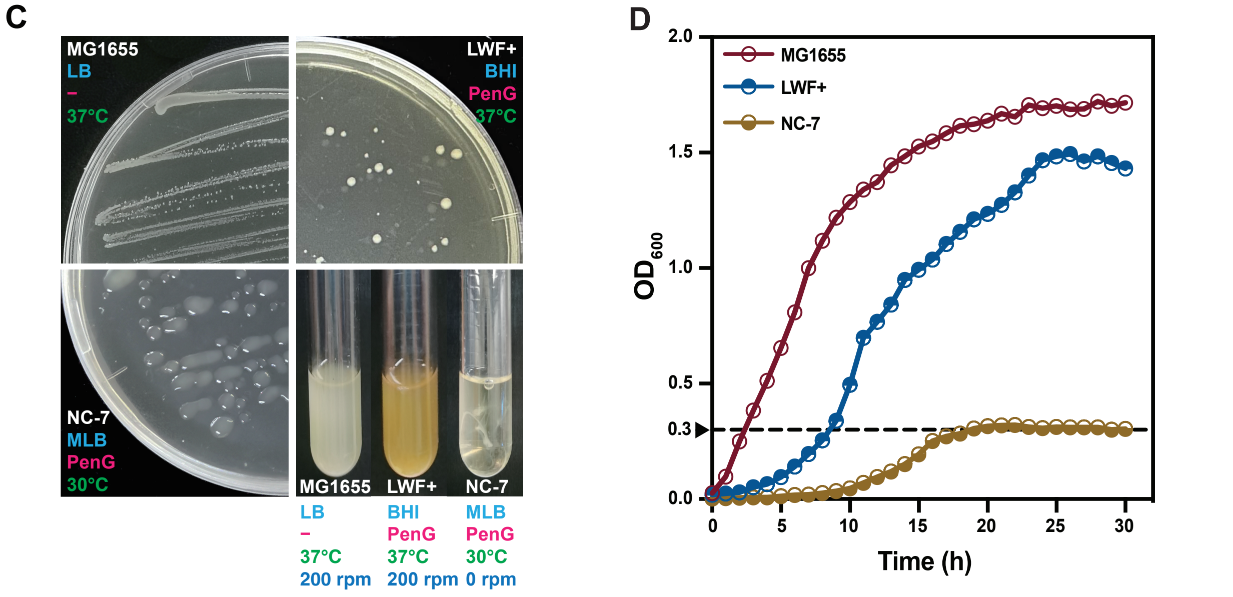

Fig. S1 Culture of two *E. coli* L-forms, NC-7 and LWF+. A Schematic diagram for generating gram-negative L-forms using cell wall-targeting reagents and laboratory experimental evolution approaches. The cell shape is transformed from rod to sphere after losing a cell wall. The final stable L-forms may hold single (OM or CM) or double (OM and CM) membranes. The information (strain origin, transform methodology, etc.) for the two studied L-form strains, NC-7 and LWF+, is listed in **B**. The images for solid and liquid culture of various *E. coli* strains, wild type (MG1655), L-forms (LWF+ and NC-7) were shown in **C**. The growth curves for all strains in liquid culture were plotted based on the optical density (OD600) as demonstrated in **D**. It is important to mention that the concentrations of the two L-forms in the liquid medium can be altered due to substantial membrane fragmentation, ultimately impacting the accuracy of the OD measurement.
