# Supplementary material for: Revealing Cell Envelope Heterogeneity in Two Stable Escherichia coli L-Forms
Source: Int J Mol Sci. 2026 Mar 30;27(7):3121. doi: 10.3390/ijms27073121 (PMC13074047; doi:10.3390/ijms27073121)
Supplement: Supplementary file 1 [file ijms-27-03121-s001.zip › Fig.S2.pdf]

Fig. S2

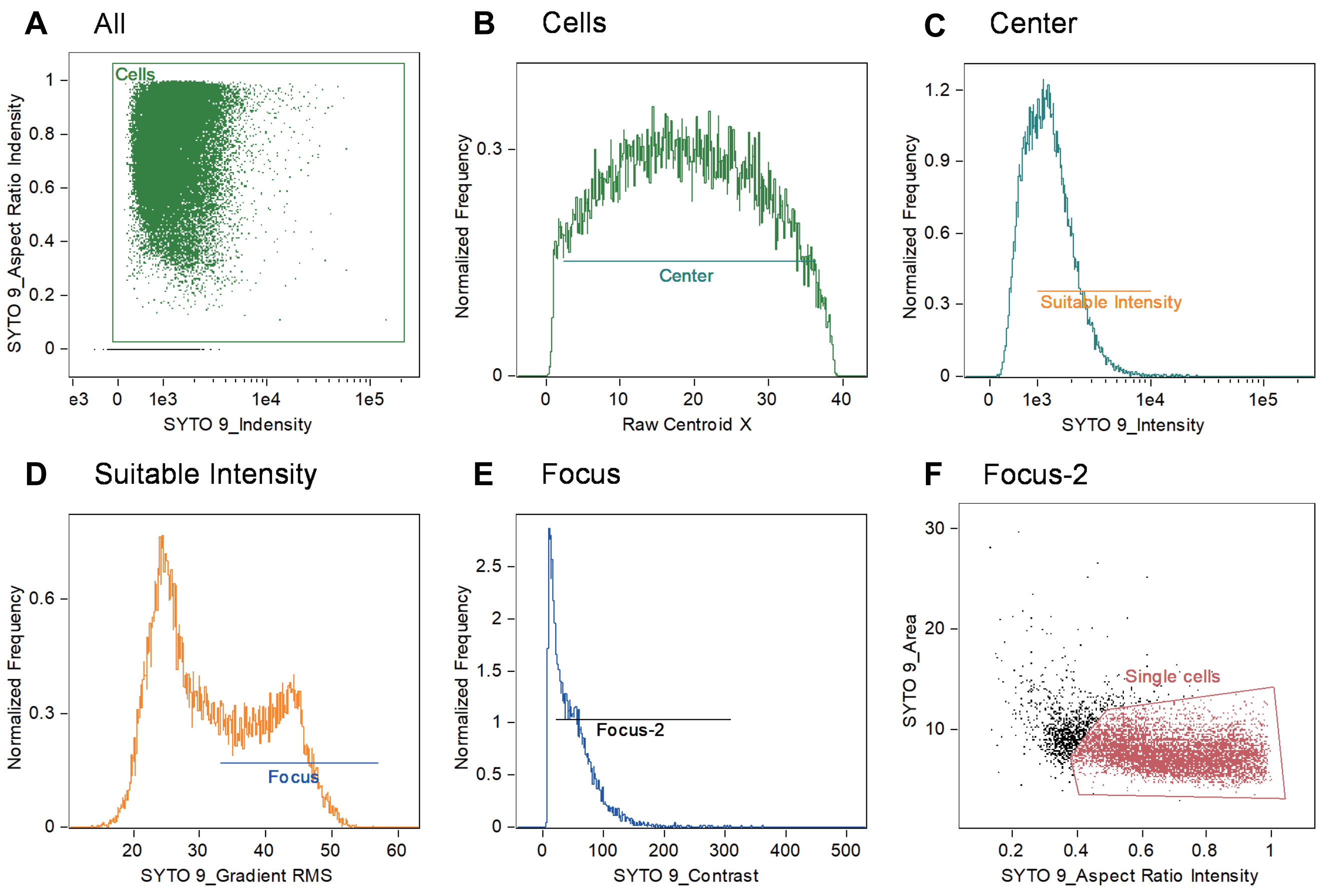

Fig. S2 Detailed procedures for imaging flow cytometry (IFC) analysis of L-form bacterial strains. Detailed protocols were established for the imaging flow cytometry (IFC)-based analysis of L-form E. coli strains. IFC data were analyzed using the IDEAS software package supplied with the instrument, which enables simultaneous visualization of individual cell images and quantitative parameters, allowing image-guided gating for accurate and high-quality data selection. The gating procedure was performed as follows:

(i) A scatter plot of fluorescence intensity from channel 2 (Intensity) versus cell aspect ratio was used to exclude SpeedBeads and cellular debris (**A**).

(ii) A histogram of the raw centroid position on the X-axis (Raw Centroid X) was examined to remove events located at the margins of the imaging field where cells were not fully captured (**B**).

(iii) A histogram of channel 2 fluorescence intensity (Intensity) was generated, and only objects with intensity values greater than 1000 were selected for further analysis (**C**).

(iv) A histogram of the gradient root-mean-square (Gradient RMS) was used to evaluate image sharpness by detecting pixel-intensity changes; well-focused cells were retained (**D**).

(v) A histogram of contrast values (Contrast)—which functions similarly to the Gradient RMS measure—was applied to further refine selection for high-clarity images (**E**).

(vi) A scatter plot of cell aspect ratio versus cell area was used, together with the corresponding cell images, to differentiate single-cell from multi-cell events (**F**).

Following these steps, a population of single, well-focused cells with appropriate fluorescence intensity was obtained. From this final population, quantitative parameters including aspect ratio, cell area, length, and width were exported for each individual cell.
